# The Perceived Impact of Length of the Diagnostic Pathway Is Associated with Health-Related Quality of Life of Sarcoma Survivors: Results from the Dutch Nationwide SURVSARC Study

**DOI:** 10.3390/cancers12082088

**Published:** 2020-07-28

**Authors:** Vicky L. M. N. Soomers, Ingrid M. E. Desar, Lonneke V. van de Poll-Franse, Michiel A. J. van de Sande, Jacco J. de Haan, Cornelis Verhoef, Ingeborg J. H. Vriens, Winan J. van Houdt, Johannes J. Bonenkamp, Winette T. A. van der Graaf, Olga Husson

**Affiliations:** 1 Department of Medical Oncology, Radboud University Medical Center, 6525 GA Nijmegen, The Netherlands; Vicky.Soomers@radboudumc.nl (V.L.M.N.S.); Ingrid.Desar@radboudumc.nl (I.M.E.D.); winette.vandergraaf@radboudumc.nl (W.T.A.v.d.G.); 2 Department of Psychosocial Research and Epidemiology, The Netherlands Cancer Institute, 1066 CX Amsterdam, The Netherlands; L.vandePoll@iknl.nl; 3 Department of Research, Netherlands Comprehensive Cancer Organization (IKNL), 3511 DT Utrecht, The Netherlands; 4 Department of Medical and Clinical Psychology, Center of Research on Psychology in Somatic Diseases, Tilburg University, CoRPS, 5037 AB Tilburg, The Netherlands; 5 Department of Orthopedics, Leiden University Medical Center, 2333 ZA Leiden, The Netherlands; M.A.J.van_de_Sande@lumc.nl; 6 Department of Medical Oncology, University Medical Center Groningen, 9713 GZ Groningen, The Netherlands; j.j.de.haan@umcg.nl; 7 Department of Surgical Oncology, Erasmus MC Cancer Institute, 3015 GD Rotterdam, The Netherlands; c.verhoef@erasmusmc.nl; 8 Department of Medical Oncology, Maastricht University Medical Centre, 6229 HX Maastricht, The Netherlands; ingeborg.vriens@mumc.nl; 9 Department of Surgical Oncology, The Netherlands Cancer Institute, 1066 CX, Amsterdam, The Netherlands; w.v.houdt@nki.nl; 10 Department of Surgical Oncology, Radboud University Medical Center, 6525 GA Nijmegen, The Netherlands; han.bonenkamp@radboudumc.nl; 11 Department of Medical Oncology, The Netherlands Cancer Institute, 1066 CX Amsterdam, The Netherlands; 12 Division of Clinical Studies, Institute of Cancer Research, London SM2 5NG, UK

**Keywords:** time to diagnosis, health-related quality of life, sarcoma, diagnostic interval

## Abstract

Background: Sarcoma patients often experience a long time to diagnosis, known as the total interval. This interval can be divided into the patient (time from symptoms to doctor consultation) and diagnostic intervals (time from first consultation to diagnosis). In other cancers, a long total interval has been associated with worse outcomes, but its effect on health-related quality of life (HRQoL) has never been investigated among sarcoma patients. This study investigates the association between (1) the actual time to diagnosis and HRQoL; (2) the perceived impact of the diagnostic interval length and HRQoL; (3) the actual length and perceived impact of the length and the HRQoL of sarcoma survivors. Methods: A cross-sectional study was performed among sarcoma patients aged ≥18, diagnosed 2–10 years ago in the Netherlands. The participants completed a questionnaire on HRQoL, the time to diagnosis, the perceived impact of the diagnostic interval on HRQoL, and coping. Results: 1099 participants were included (response rate, 58%). The mean time since diagnosis was 67.4 months. More than half reported a patient (60%) or diagnostic interval (55%) ≥1 month. A third (31%) perceived a negative impact of the diagnostic interval length on HRQoL. Patient or diagnostic interval length was not associated with HRQoL. By contrast, participants perceiving a negative impact (32%) had lower HRQoL scores than those perceiving a positive (11%) or no impact (58%) (*p* = 0.000). This association remained significant in a multivariable model, in which maladaptive coping strategies and tumour characteristics were also found to be associated with HRQoL. Participants perceiving a negative impact of the length of the diagnostic interval related this to high psychological distress levels, more physical disabilities, and worse prognosis. Conclusion: The perceived impact of the diagnostic interval length was associated with the HRQoL of sarcoma survivors, whereas the actual length was not associated with HRQoL. Maladaptive coping strategies were independently associated with HRQoL. This offers opportunities for early intervention to improve HRQoL.

## 1. Introduction

Sarcomas are mesenchymal tumours, with considerable heterogeneity regarding the age of onset, anatomic location, histological subtype, and outcome. These solid tumours consist of more than 100 histologic subtypes that originate in soft tissue (STS; 80%) or bone (BS; 20%). Sarcomas are typical examples of rare cancers (affecting fewer than six individuals per 100,000/year) and have an estimated incidence of 4–5 per 100,000 per year [1]. Patients with rare cancers have worse outcomes than patients with common cancers: in the Netherlands, the five-year survival rates for all subtypes of STS and BS taken together are 58% and 49%, respectively, which are lower than the five-year survival rates for all cancers diagnosed in the Netherlands (65%) [2]. Given the rarity and heterogeneity of the disease, sarcomas are often not recognized by healthcare providers, leading to delayed diagnostic pathways. Additionally, the diagnostic process can be complex, leading to a prolonged diagnostic time in expert centres. Furthermore, a lack of expert pathologists and the absence of tumour-specific multidisciplinary teams, cancer-specific therapies, and clinical trials often preclude patients with rare cancer from receiving a proper, timely diagnosis and adequate care [3].

Historically, the evaluation of oncologic care has focused on clinical outcomes, such as treatment-related toxicities and overall survival. Currently, more attention is being given to patient-reported outcomes, such as health-related quality of life (HRQoL). HRQoL refers to the impact of disease and treatment on domains of physical, psychological, and social functioning [4]. In other malignancies, a prolonged time to cancer diagnosis has been associated with worse HRQoL [5]. The association between the time to diagnosis and HRQoL has never been investigated quantitatively among sarcoma patients, but qualitative reports indicate that the time to diagnosis influences patients’ physical and psychosocial well-being [6,7]. This may not only influence HRQoL in the short-term but may also influence HRQoL among sarcoma survivors.

Although the survival rates of sarcoma patients lag behind those of common cancer patients, there are an estimated 280,000 sarcoma survivors in Europe [1] who require supportive and rehabilitation services. To improve these services, we need to understand the care experiences and needs of sarcoma survivors. Survivorship care focusses on the health and well-being of a person with cancer from the time of diagnosis until the end of life [8], including issues related to follow-up care (e.g., regular health and wellness check-ups), late effects of treatment, cancer recurrence, second cancers, and HRQoL. 

This study investigates the association between actual time to diagnosis and HRQoL in a group of adult sarcoma survivors. Furthermore, the perceived impact of the diagnostic interval length on HRQoL, both quantitative and qualitative, and the independent association of time to diagnosis and other variables with HRQoL are investigated. 

## 2. Methods

### 2.1. Study Design and Participants

This cross-sectional cohort study included Dutch sarcoma patients aged ≥18, registered in the Netherlands Cancer Registry (NCR), and diagnosed between 1 January 2008 and 31 December 2016 at one of the participating sarcoma expertise centres (Radboud University Medical Centre Nijmegen, Antoni van Leeuwenhoek Amsterdam-The Netherlands Cancer Institute, University Medical Centre Groningen, Leiden University Medical Centre, Erasmus MC Cancer Institute Rotterdam, and Maastricht University Medical Centre). Participants had to be able to complete Dutch questionnaires by themselves. Patients with desmoid fibromatosis, grade 1 chondrosarcoma, atypical lipomatous tumours, giant-cell tumours, or gastro-intestinal stromal tumours were excluded due to their indolent clinical behaviour or different treatment strategies compared to patients with other sarcomas. Ethical approval was given by the medical ethical committee of Radboud University Medical Centre (2017-3944), and the study was registered in the Dutch Trial Registry (NTR-7253). 

### 2.2. Recruitment and Data Collection

Eligible patients received a letter from their (ex-)treating physician explaining the purpose of the study. After providing informed consent, patients could complete questionnaires either online or by pencil-and-paper. Further details of this method have been described previously [9,10].

### 2.3. Study Measures

While this study was primarily designed to examine HRQoL among sarcoma survivors compared to that in an age- and sex-matched normative population (https://www.trialregister.nl/trial/7048; NTR-7253, accessed on 24 July 2020), the current study is a preplanned, secondary analysis investigating the association between time to diagnosis and HRQoL.

### 2.4. Time to Diagnosis

The time to diagnosis is often referred to as the total interval. The total interval, describing the time from the first symptom until (histological) diagnosis, can be divided into patient and diagnostic intervals [6,11,12]. These encompass the time from the first symptom until the first presentation to a doctor (patient interval), and the time from this first presentation until pathologic diagnosis (diagnostic interval). The diagnostic interval can be further divided into primary care, secondary care, and tertiary care intervals.

Questions on patient and diagnostic interval length were designed by the study group, all the intervals were patient-reported, and the answers were categorical. The meaning of each interval was explained in detail to the patients to overcome interpretation issues. The study population was grouped by the length of the patient and diagnostic intervals, with a cut-off point of 1 month, based on previous literature [13,14,15,16]. Many countries deem four weeks or one month for the diagnostic interval as appropriate, and the Dutch SONCOS guidelines (Stichting ONCologische Samenwerking; foundation for multidisciplinary oncological collaboration) accepts a period of four weeks between referral by the GP and histological cancer diagnosis [17].

### 2.5. Health-Related Quality of Life: EORTC QLQ-C30

HRQoL was measured by the European Organization for Research and Treatment for Cancer Quality of Life Questionnaire-C30 (EORTC QLQ-C30). This self-administered 30-item questionnaire has been validated to measure HRQoL in cancer patients [18]. It consists of global quality of life (QoL), five functional scales, three symptom scales, and several single items assessing additional symptoms and the perceived financial impact of the disease. The global QoL is the patient’s rating of their overall health and quality of life during the past week; the functional scales assess physical, cognitive, role, social, and emotional functioning. The global QoL and the functioning scales were used in our analyses. After the linear transformation of the raw scores, all the scores range from 0 to 100; a higher score represents a better global QoL or level of functioning [19]. Apart from a quantitative score, one can examine clinical relevance using the guidelines of Cocks et al.; they distinguish four effect sizes: trivial, small, medium, and large [20]. Each scale has its own threshold between size classes. A guideline for the interpretation of the clinical impact of emotional functioning is missing; therefore, the cut-off points of role functioning were used, as this is the most conservative scale and this method has been described previously [21].

### 2.6. Perceived Impact of Diagnostic Interval Length on HRQoL

This was assessed by a single question—“Do you think your HRQoL was influenced by your diagnostic interval length?”—and the option for patients to explain their multiple-choice answer (yes, negatively; yes, positively; or no) in an open text field.

### 2.7. Socio-Demographic and Tumour Characteristics

Tumour characteristics were available from the NCR, which routinely collects data of all individuals newly diagnosed with cancer. These include the date of diagnosis, histology, tumour grade, localisation, and stage at diagnosis, as well as several patient characteristics such as the age at diagnosis, gender, and socio-economic status (SES). Patients were grouped into clinically relevant subgroups based on age (18–39, 40–69, ≥70 years old). SES was derived from zip codes and is based on education, income, and employment status [22]. The date of participation was subtracted from the date of diagnosis to calculate the time since diagnosis. The treatment modalities were patient-reported and include all the treatments patients have had since their primary diagnosis.

### 2.8. Coping

Coping, the way in which an individual conducts themself to decrease the effect of a stressful situation [23], was assessed using the Illness Cognition Questionnaire for chronic diseases (ICQ) [24]. This 18-item questionnaire consists of three cognition subscales of six items rated on a 4-point Likert scale: helplessness as a way of emphasizing the aversive meaning of the disease, acceptance as a way of diminishing the aversive meaning, and perceived benefits as a way of adding a positive meaning to the disease [24]. The total subscale scores could range from 6 to 24, with higher scores on helplessness and lower scores on acceptance and disease benefits indicating maladaptive coping strategies. Adaptive coping means that one evaluates the situation, actively seeks help, considers all thinkable solutions, and actively tries to solve the problem, while maladaptive coping means one will try to move away from the stressful event, indicating that the problem will not be solved.

### 2.9. Statistical Analyses

Descriptive statistics were used to describe the study population. Categorical variables are presented as numbers and percentages; for continuous variables, means and standard deviations are reported.

To investigate the association between the patient and diagnostic interval length, perceived impact of the length of the diagnostic interval, and HRQoL (global QoL and all five functioning scales), a series of one-way analysis of variance (ANOVA) tests were conducted, with a cut-off point of 1 month.

The independent association between the interval length, perceived impact of the diagnostic interval on HRQoL, patient and tumour characteristics, and coping strategies were tested using multivariable linear regression analyses (for global QoL and all five functioning scales).

Open field answers given by patients who described a negative impact of their diagnostic interval length on HRQoL were analysed by two investigators (Vicky Soomers and Olga Husson using inductive coding, followed by axial coding to define the main themes. Quotes to illustrate the results were selected.

Missing items in the multi-item domains of the EORTC-QLQ-C30 were imputed with simple mean imputation, according to the guidelines of the EORTC Quality of Life Group [19]. After the imputation of these values, an available cases analysis was performed. All other missing data were assumed to be missing at random, and only available data were analysed. All statistical analyses were performed using IBM SPSS 25.0 (IBM, Chicago, USA); two-sided *p*-values of <0.05 were considered statistically significant.

## 3. Results

### 3.1. Participants

We included 1099 of the 1887 invited sarcoma patients (response rate, 58%). The flow-chart and characteristics of the responders have been published before [9]. Responders were diagnosed at a mean age of 54.6 years, 54% were male, the mean time since diagnosis was 67.4 months, 76% were diagnosed with STS, and 47% of the sarcomas were located in the extremities (Table 1).

### 3.2. Health-Related Quality of Life by Patient and Diagnostic Interval Length

**Figure 1:**
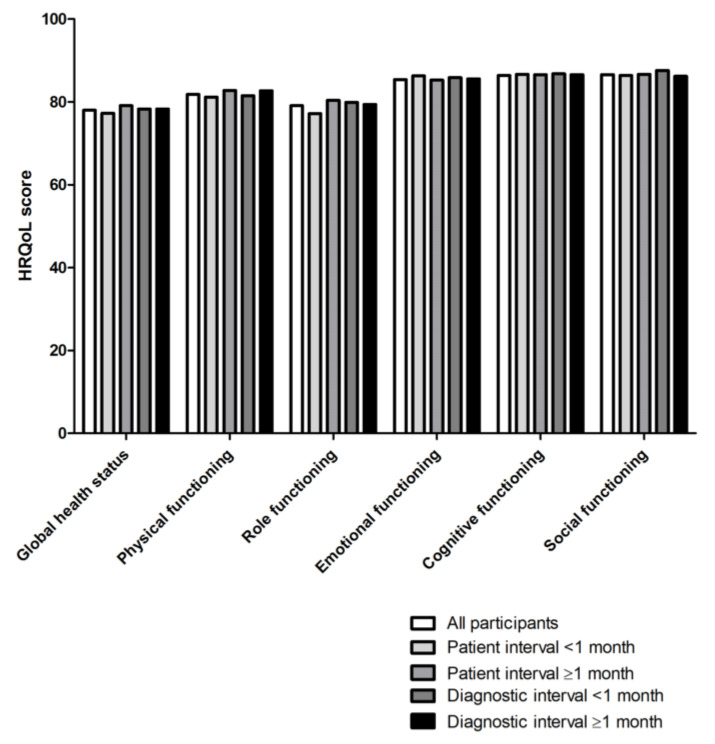
Mean scores on HRQoL.

The patient interval lasted ≥1 month in 60% (*n =* 589). The diagnostic interval lasted ≥1 month in 55% (*n =* 569). No statistical differences in HRQoL were found for the different patient and diagnostic interval groups (Figure 1).

### 3.3. Influence of Perceived Impact of Diagnostic Interval on HRQoL

More than half of the participants (58%, *n =* 620) thought their HRQoL was not influenced by their diagnostic interval length, whilst 31% (*n =* 337) and 11% (*n =* 115) thought their HRQoL was influenced negatively or positively by their diagnostic interval length, respectively.

**Figure 2:**
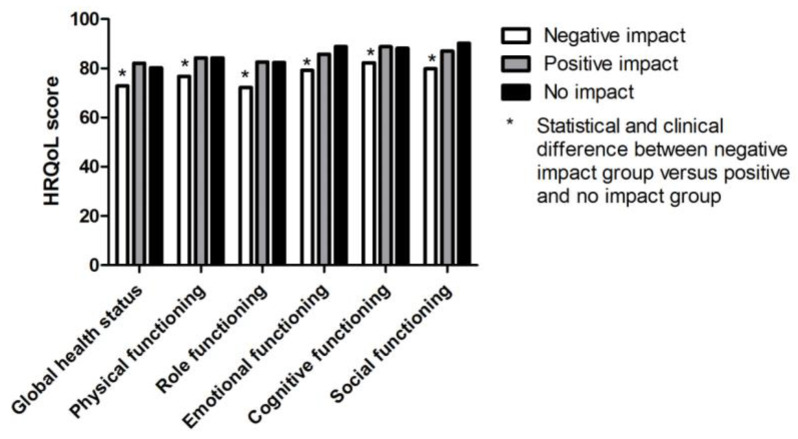
Mean HRQoL scores by impact of diagnostic interval on HRQoL.

In all domains, patients with a perceived negative impact of the diagnostic interval length on HRQoL scored significantly lower than the patients with no or a positive perceived impact. (Figure 2). All these differences showed a small clinically relevant difference as well, both between the groups who perceived a negative impact versus the group experiencing a positive impact or no impact. There was no difference between the group experiencing a positive impact and those experiencing no impact.

### 3.4. Independent Association of Patient Interval and Patient and Tumour Characteristics with HRQoL

Global QoL was independently associated with the perceived impact of the diagnostic interval length on HRQoL (Table 2). Participants perceiving a positive or no impact had a higher global QoL than those perceiving a negative impact. Furthermore, participants with a lower global QoL score showed maladaptive coping strategies, with higher scores on helplessness and lower scores on acceptance and disease benefits. Several tumour characteristics were associated with global QoL.

Similar results were found for the functioning scales (Table 2). Coping strategies and especially the helplessness scale were negatively associated with all the functioning scales. The perceived impact of the diagnostic interval length on HRQoL showed a similar trend for all the functioning scales but was only significant for the difference between the no impact group and the negative impact group on the emotional and social functioning scale. Age was associated with all the functioning scales, and gender, SES, and time since diagnosis were associated with physical functioning.

### 3.5. Independent Association of Diagnostic Interval and Patient and Tumour Characteristics with HRQoL

Although diagnostic interval length was not associated with global QoL, the perceived impact of the diagnostic interval length remained significantly associated (Table 3). Participants perceiving a positive or no impact had a higher global QoL. Those with maladaptive coping strategies had lower global QoL scores. Both location and histology were associated with global QoL in the model including patient interval length.

On the functioning scales, coping strategies, especially the helplessness and acceptance scales, remained independently associated. The age at diagnosis, gender, SES, and a longer time since diagnosis remained associated as well.

### 3.6. Consideration of Patients Who Perceive a Negative Impact of Their Diagnostic Interval Length on HRQoL; Qualitative Analyses

We identified three main themes: psychological distress, physical inability, and an influence on prognosis. Of the patients who commented on why their HRQoL was influenced negatively by their diagnostic interval length (*n =* 298), 52% said this was due to psychological distress: an increase in insecurity, fear, and stress. Of these participants, 73% had a diagnostic interval length ≥1 month, and 39%, ≥3 months. They experienced these emotions not only during the diagnostic trajectory but also in their current lives. They feared the recurrence of disease, metastases, or death.

- “Fear, you don’t know when and where it recurs. You continuously monitor your body.”
- “Heavy psychological stress during the diagnostic trajectory.”

Many (41%) reported more physical inability due to longer lasting complaints, the growth of the tumour, and, consequently, more elaborate treatment, such as larger operations and the addition of radiotherapy or chemotherapy. Of these participants, 81% reported a diagnostic interval ≥1 month, and 63%, ≥3 months. Some (7%) thought it influenced their prognosis and thought metastases or disease recurrence could have been avoided with an earlier diagnosis and would have possibly led to a curative treatment. Of these, 86% experienced a diagnostic interval ≥1 month and 36%, ≥3 months.

- “A lot of pain longer than necessary. Surgical intervention was not possible anymore due to the long diagnostic trajectory.”
- “Then they would not have to cut it out this far, so I would have fewer complaints now.”
- “Yes, because if there had been an earlier intervention, then the sarcoma would not have been this large and I would not have had metastases.”
- “Due to not tackling it immediately it came back twice.”

## 4. Discussion

In this cross-sectional cohort study among a large sarcoma survivorship population, we found that patient and diagnostic interval length were not associated with HRQoL scores, but the perceived impact of the diagnostic interval length on HRQoL was associated with HRQoL scores.

There have been no published studies looking at the effect of patient and diagnostic interval length on HRQoL among cancer survivors. However, in a systematic review about the effect of total interval length on the outcomes for symptomatic cancer just after diagnosis, Neal et al. found that an earlier diagnosis of cancer will likely improve HRQoL [5].

Although patient and diagnostic interval length were not associated with HRQoL scores, we found the perceived impact of the diagnostic interval length on HRQoL to be independently associated with global QoL and several HRQoL functioning scales. Participants perceiving a negative impact had lower HRQoL scores than those perceiving a positive or no impact. Furthermore, participants with lower HRQoL scores used maladaptive coping strategies. Participants perceiving a negative impact of their diagnostic interval on their HRQoL showed higher scores on the subscale helplessness and lower scores on the acceptance and disease benefits scales. Both the perceived impact and coping remained independently associated in our multivariable model. The question remains why perceived impact remains associated; this may be due to the actual time to diagnosis, or other patient or tumour characteristics. The coping strategy was also found to be a significant predictor of HRQoL among other malignancies [25,26,27]. The use of coping strategies can vary between patients and over time or between situations [23]. Although probably not sarcoma specific and solely related to the diagnostic interval length, our results indicate that supportive services focusing on developing adaptive coping strategies may positively influence patients’ HRQoL.

Our finding that the perceived impact of the diagnostic interval length on HRQoL is associated with HRQoL scores is further supported by the qualitative analysis of the open text field answers, in which many patients perceiving a negative impact describe psychological distress, more physical disabilities, and a worse prognosis due to their diagnostic interval length. Although their perception of the influence of the relationship between prognosis and diagnostic interval length may not be entirely supported by current literature [6], it is worrying that 2–10 years after diagnosis, patients still report this psychological and physical burden. We are not aware of previous studies examining patients’ perception of diagnostic interval length and HRQoL. However, our findings are supported by two British and two Danish studies, which reported that cancer patients diagnosed through fast-track referrals were less likely to be dissatisfied with the length of waiting times and more likely to be satisfied with their subsequent cancer care than those referred electively [28,29,30,31].

To our knowledge, this is the only study that has investigated the influence of patient and diagnostic interval length and the perceived impact of the diagnostic interval length on HRQoL among adult sarcoma survivors. The overall completeness of the variables in our analysis sample was high.

Our study has several limitations. First, although we invited all patients diagnosed with sarcoma in the Netherlands between 2008 and 2016, there was a selection bias with a probable overrepresentation of patients with high health literacy. Furthermore, due to the survivorship nature of the study, there is a natural selection of patients with favourable prognoses, less aggressive histological subtypes, and low co-morbidity. The longitudinal assessment, preferably in daily clinical practice, of HRQoL, starting from diagnosis throughout the disease continuum, will provide more comprehensive data to determine causal associations and will be less prone to recall bias. We have set up a prospective study in which newly diagnosed sarcoma patients are asked to complete questionnaires for a period of two years (QUEST study; ClinicalTrials.gov Identifier: NCT03441906) to overcome this limitation. Second, our interval length data are subject to recall bias. However, 90% and 95% of the patients indicated they still remembered their patient and diagnostic interval lengths. In general, significant events, such as cancer diagnosis, are not likely to be forgotten [32]. Furthermore, the estimation of the duration of an event is extremely stable [33]. Third, one may argue that a cut-off of 1 month for the diagnostic interval length for sarcomas is too short. However, a sensitivity analyses with a cut-off of 3 months did not show different results. Last, one of the biggest challenges when measuring patient-reported outcomes is determining what instrument assesses HRQoL or other relevant topics best among the study population. Given the qualitative results in our data, the EORT-QLQ-C30 may be too generic to measure the impact of the total interval length on the HRQoL of sarcoma survivors. In future research, patient-reported outcome measures focusing on disability, distress, or recurrence may better capture this relationship.

Our study has resulted in more understanding of the survivorship experience. The perceived impact of the diagnostic interval and coping strategies have a long-lasting effect on global HRQoL and all the functional scales; it thus seems important to keep the diagnostic trajectory and perception thereof as short as possible. Strategies could include creating awareness among the general population and healthcare providers about sarcoma, reference networks, and centres of expertise, with rapid diagnostic pathways at sarcoma centres.

Since the perceived effect of diagnostic interval length still causes physical and psychological disabilities amongst patients 2–10 years after diagnosis, the improvement of services, treatment, and rehabilitation programs may contribute to improving the HRQoL of sarcoma patients. Patients with maladaptive coping strategies are at risk of lower HRQoL. Sarcoma care could be improved if healthcare providers acknowledge patients’ frustrations regarding their diagnostic pathway and have an eye for their coping strategies. If necessary, supportive care focusing on coping strategies could be given early in the treatment pathway. This will improve HRQoL and may lead to more personalized rehabilitation services, probably resulting in the restoration of social and employment activities and thus having a positive economic impact.

## 5. Conclusions

The perceived impact of the diagnostic interval length was associated with the HRQoL of sarcoma survivors, whereas the actual length was not associated with HRQoL. Maladaptive coping strategies were independently associated with HRQoL. The sarcoma diagnostic pathway should be kept short in order to provide the best treatment possible and optimize HRQoL.

## Figures and Tables

**Table 1:** Participant characteristics.

	All Patients (*n =* 1099)
Gender *n* (%)	
Male	595 (54)
Female	504 (46)
Age at diagnosis in years: Mean (SD)	54.6 (15.4)
Socio-economic status *n* (%)	
Low	286 (26.1)
Intermediate	462 (42.1)
High	349 (31.8)
Coping: mean (SD)	
Helplessness	8.8 (3.6)
Acceptance	18.8 (4.2)
Disease benefits	15.8 (4.8)
Time since diagnosis in months: Mean (SD)	67.4 (30.4)
Location *n* (%)	
Extremities	514 (47)
Non-extremities	585 (53)
Histology *n* (%)	
Soft tissue sarcoma	835 (76)
Dermatofibrosarcoma protuberans	74 (7)
Liposarcoma	177 (16)
Myxofibrosarcoma	137 (13)
Leiomyosarcoma	113 (10)
Rhabdomyosarcoma	15 (1)
Malignant peripheral nerve sheath tumors (MPNST)	34 (3)
Synovial sarcoma	35 (3)
Vascular sarcoma	43 (4)
Other soft tissue sarcoma	201 (18)
Bone sarcoma	264 (24)
Osteosarcoma	69 (6)
Chondrosarcoma	130 (12)
Chordoma	30 (3)
Ewing sarcoma	28 (3)
Other bone sarcoma	13 (1)
Grade *n* (%)	
Low grade	614 (60)
Intermediate or high grade	407 (40)
Metastases at diagnosis *n* (%)	
Not present	1067 (97)
Present	32 (3)
Treatment modalities *n* (%)	
Surgery	448 (43)
Surgery and radiotherapy	422 (41)
Surgery and chemotherapy	79 (8)
Surgery and radiotherapy and chemotherapy	86 (8)

The *n* of an individual cell may be smaller due to missing values. Participants who had only undergone radiotherapy, chemotherapy, or a combination of the two were excluded from this analysis, due to their small group size and unreliable analysis.

**Table 2:** Standardized betas of multivariable linear regression analysis evaluating the association of the patient interval and other variables with EORTC QLQ-C30 scales.

	Global QoL	Physical Functioning	Role Functioning	Emotional Functioning	Cognitive Functioning	Social Functioning
**Patient interval**						
<1 month	ref	ref	ref	ref	ref	ref
≥1 month	0.031	0.017	0.047	–0.032	–0.006	–0.008
**Perceived impact of diagnostic interval length**						
–negative	ref	ref	ref	ref	ref	ref
–positive	0.088 **	0.040	0.044	0.026	0.051	0.027
–no impact	0.065 *	0.032	0.034	0.092 **	0.044	0.086 **
**Gender**						
–male	ref	ref	ref	ref	ref	ref
–female	–0.038	–0.064 *	0.009	–0.039	–0.042	–0.010
**Age at diagnosis**	–0.018	–0.231 **	–0.086 **	0.174 **	0.103 **	0.071 *
**Socio–economic status**						
–low	ref	ref	ref	ref	ref	ref
–intermediate	0.029	0.060*	0.007	0.014	–0.019	0.018
–high	0.034	0.062*	0.029	0.020	0.044	0.038
**Coping**						
–helplessness	–0.441 **	–0.601 **	–0.601 **	–0.386 **	–0.323 **	–0.518 **
–acceptance	0.157 **	–0.005	0.063 *	0.239 **	0.113 **	0.122 **
–disease benefits	0.118 **	0.017	0.030	0.010	0.013	0.011
**Time since diagnosis**	–0.036	–0.064 **	–0.035	0.042	0.005	–0.006
**Location**						
–non–extremity	ref	ref	(0.0 ref)	ref	ref	ref
–extremity	0.081 **	–0.060 *	0.037	0.049	0.106 **	0.045
**Histology ^#^**						
–dermatofibrosarcoma	ref	ref	ref	ref	ref	ref
–liposarcoma	–0.103 *	0.041	0.063	0.12	0.061	0.000
–myxofibrosarcoma	–0.135 **	0.013	0.013	–0.025	–0.020	–0.035
–leiomyosarcoma	–0.058	0.036	0.087 *	–0.026	–0.018	0.040
–rhabdomyosarcoma	0.011	0.011	0.042	0.018	0.031	0.044
–malignant peripheral nerve sheath tumors (MPNST)	0.000	–0.065 *	–0.005	0.010	0.002	0015
–synovial	–0.037	0.015	–0.014	0.013	0.019	0.002
sarcoma						
–vascular	–0.014	–0.019	–0.001	–0.020	0.023	–0.006
sarcoma						
–other STS	–0.132 *	0.041	0.049	0.078	0.028	0.034
–osteosarcoma	–0.053	–0.098 **	–0.049	0.067	0.026	0.026
–chondrosarcoma	–0.075	–0.048	–0.041	0.061	0.010	0.011
–chordoma	–0.125 **	–0.102 **	–0.042	–0.008	0.003	–0.046
–Ewing sarcoma	–0.012	–0.033	0.010	0.080 *	–0.002	0.028
–other BS	–0.042	–0.024	0.003	0.007	0.026	0.014
**Grade**						
–low	ref	ref		ref	ref	ref
–high	–0.059	–0.055		–0.044	–0.011	–0.014
**Metastases at diagnosis**						
–not present	ref	ref	ref	ref	ref	ref
–present	–0.037	0.027		–0.016	–0.054	–0.038
**Treatment modality**						
–surgery plus	ref	ref	ref	ref	ref	ref
CHx, RTx, or both						
–surgery alone	–0.009	–0.002		0.000	0.079 *	0.028

^#^ STS = soft tissue sarcoma; BS = bone sarcoma; CHx = chemotherapy; RTx = radiotherapy. * *p <* 0.05. ** *p <* 0.01.

**Table 3:** Standardized betas of multivariable linear regression analysis evaluating the association of the diagnostic interval and other variables with EORTC QLQ-C30 scales.

	Global QoL	Physical Functioning	Role Functioning	Emotional Functioning	Cognitive Functioning	Social Functioning
**Diagnostic interval**						
<1 month	ref	ref	ref	ref	ref	ref
≥1 month	0.012	0.016	–0.015	0.019	0.012	–0.027
**Perceived impact of diagnostic interval length**						
–negative	ref	ref	ref	ref	ref	ref
–positive	0.087 **	0.042	0.035	0.034	0.055	0.021
–no impact	0.066 *	0.035	0.027	0.099 **	0.048	0.079 *
**Gender**						
–male	ref	ref	ref	ref	ref	ref
–female	–0.037	–0.064 **	0.011	–0.041	–0.043	–0.009
**Age at diagnosis**	–0.018	–0.230 **	–0.089 **	0.177 **	0.104 **	0.069 *
**Socio–economic status**						
–low	ref	ref	ref	ref	ref	ref
–intermediate	0.029	0.060 *	0.007	0.015	–0.019	0.018
–high	0.035	0.062 *	0.03	0.019	0.044	0.038
**Coping**						
–helplessness	–0.441 **	–0.600 **	–0.604 **	–0.384 **	–0.322 **	–0.520 **
–acceptance	0.157 **	–0.005	0.064 *	0.238 **	0.113 **	0.122 **
–disease benefits	0.117 **	0.017	0.027	0.012	0.014	0.01
**Time since diagnosis**	–0.036	–0.066 **	–0.035	0.042	0.005	–0.007
**Location**						
–non–extremity	ref	ref	ref	ref	ref	ref
–extremity	0.083 **	–0.059 *	0.04	0.046	0.105 **	0.045
**Histology ^#^**						
–dermatofibrosarcoma	ref	ref	ref	ref	ref	ref
–liposarcoma	–0.110 *	0.037	0.054	0.017	0.061	0.003
–myxofibrosarcoma	–0.141 **	0.009	0.005	–0.02	–0.02	–0.032
–leiomyosarcoma	–0.062	0.033	0.083 *	–0.024	–0.018	0.043
–rhabdomyosarcoma	0.008	0.009	0.038	0.021	0.031	0.045
–malignant peripheral nerve sheath tumors (MPNST)	–0.003	–0.067*	–0.007	0.01	0.002	0.017
–synovial	–0.039	0.013	–0.012	0.011	0.017	0.005
**sarcoma**						
–vascular	–0.018	–0.022	–0.006	–0.017	0.023	–0.004
**sarcoma**						
–other STS	–0.139 **	0.037	0.04	0.084	0.029	0.038
–osteosarcoma	–0.055	–0.100 **	–0.051	0.068	0.026	0.028
–chondrosarcoma	–0.079	–0.051	–0.042	0.061	0.009	0.015
–chordoma	–0.127 **	–0.103 **	–0.042	–0.008	0.003	–0.044
–Ewing sarcoma	–0.013	–0.034	0.008	0.081 *	–0.001	0.029
–other BS	–0.044	–0.025	–0.001	0.009	0.026	0.014
**Grade**						
–low	ref	ref	ref	ref	ref	ref
–high	–0.058	–0.054	–0.011	–0.044	–0.01	–0.015
**Metastases at diagnosis**						
–not present	ref	ref	ref	ref	ref	ref
–present	–0.037	0.027	0.005	–0.017	–0.054	–0.037
**Treatment modality**						
–surgery plus	ref	ref	ref	ref	ref	ref
CHx, RTx, or both						
–surgery alone	–0.006	–0.001	0.037	–0.003	0.078*	0.028

^#^ STS = soft tissue sarcoma; BS = bone sarcoma; CHx = chemotherapy; RTx = radiotherapy. * *p <* 0.05. ** *p <* 0.01.
